# Supplementary material for: Splice-Junction-Based Mapping of Alternative Isoforms in the Human Proteome
Source: Cell Rep. Author manuscript; Available in PMC 2020 Jan 15. (PMC6961840; doi:10.1016/j.celrep.2019.11.026)
Supplement: 2 [file NIHMS1546469-supplement-2.zip › DF1/PXD006675/VentricularSeptum/VentricularSeptum_7_TTN_VEAEPAEEVTIMEEK.pdf]

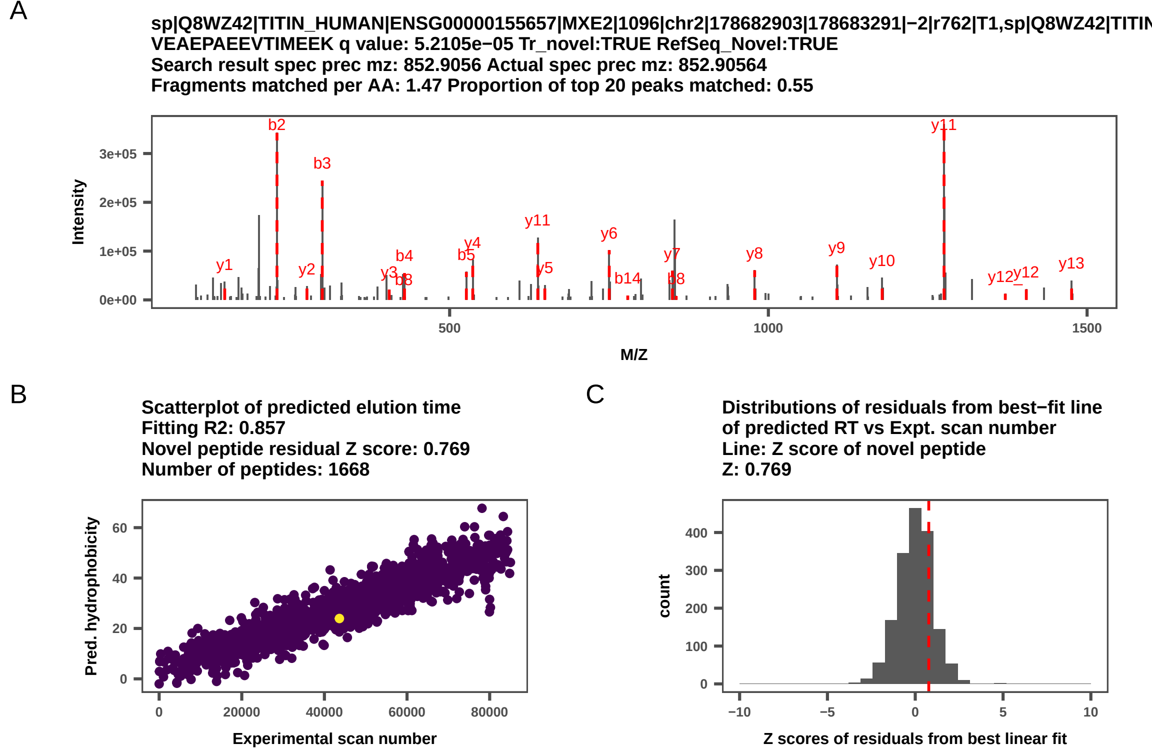
A
sp|Q8WZ42|TITIN_HUMAN|ENSG00000155657|MXE2|1096|chr2|178682903|178683291|−2|r762|T1,sp|Q8WZ42|TITIN
VEAEPAEEVTIMEEK q value: 5.2105e−05 Tr_novel:TRUE RefSeq_Novel:TRUE
Search result spec prec mz: 852.9056 Actual spec prec mz: 852.90564
Fragments matched per AA: 1.47 Proportion of top 20 peaks matched: 0.55
Intensity
3e+05
2e+05
1e+05
0e+00
b2
b3
y1
y2
y3
b8
b4
b5
y4
y11
y5
y6
b14
y7
b8
y8
y9
y10
y11
y12
y12
y13
500
1000
1500
M/Z
B
Scatterplot of predicted elution time
Fitting R2: 0.857
Novel peptide residual Z score: 0.769
Number of peptides: 1668
Pred. hydrophobicity
60
40
20
0
0
20000
40000
60000
80000
Experimental scan number
C
Distributions of residuals from best−fit line
of predicted RT vs Expt. scan number
Line: Z score of novel peptide
Z: 0.769
count
400
300
200
100
0
−10
−5
0
5
10
Z scores of residuals from best linear fit
